# 20-Hydroxyecdysone Modulates Bmp53-Mediated Apoptosis Regulation by Suppressing Mdm2-like-Dependent Ubiquitination in Silkworm, *Bombyx mori*

**DOI:** 10.3390/insects16050544

**Published:** 2025-05-21

**Authors:** Meixian Wang, Dingding Han, Luyang Xiang, Jiahao Wang, Benteng Luo, Xingjia Shen

**Affiliations:** 1 Jiangsu Key Laboratory of Sericultural and Animal Biotechnology, School of Biotechnology, Jiangsu University of Science and Technology, Zhenjiang 212100, China; mxwang@just.edu.cn (M.W.); 18852804604@163.com (D.H.);; 2 Key Laboratory of Silkworm and Mulberry Genetic Improvement, Ministry of Agriculture and Rural Affairs, The Sericultural Research Institute, Chinese Academy of Agricultural Sciences, Zhenjiang 212100, China

**Keywords:** apoptosis, Bmp53, Mdm2-like, ubiquitination, 20E

## Abstract

In the silkworm, 20-hydroxyecdysone (20E) induces apoptosis and autophagy, driving larval organ degeneration and remodeling. This mechanism may be a potential target for eco-friendly strategy for insect pests. However, a major challenge in harnessing this approach lies in the insufficient understanding of 20E’s regulatory process in such a cell death mechanism. Our previous research has identified *Bmp53* as a crucial gene in promoting the development of *Bombyx mori* during the pupal stage by inducing apoptosis, and has predicted the potential apoptotic regulatory network of Bmp53, wherein Mdm2-like ubiquitinating structural protein serves as a key component of this network. This study demonstrates that Mdm2-like acts as a ubiquitination regulatory protein, controlling its apoptosis-inducing activity via interaction with Bmp53. Moreover, co-expression of Mdm2-like and Bmp53 indirectly affects gene expression in the 20E-mediated apoptosis pathway. Further investigation revealed that Mdm2-like suppresses 20E-induced apoptosis by downregulating Bmp53 expression. This study reveals that the ubiquitination-mediated Mdm2-like/Bmp53 apoptosis pathway is a novel mechanism regulating silkworm apoptosis, with 20E playing a crucial role in this process. These findings enhance our understanding of the genetic mechanisms underlying tissue degradation during the metamorphic stage of the *Bombyx mori.* Additionally, these insights provide a theoretical reference for the development of environmentally friendly, hormone-based control strategies targeting protein modification as a means of managing lepidopteran pests.

## Simple Summary

This study elucidates the MDM2-like–Bmp53 ubiquitination axis as a novel apoptotic regulatory mechanism in *Bombyx mori* and potentially other *Lepidoptera* species. Importantly, we demonstrate the critical involvement of 20-hydroxyecdysone (20E) signaling in modulating this pathway, establishing a molecular framework for understanding the ubiquitination-dependent regulation of 20E-mediated metamorphosis and developmental processes.

## 1. Introduction

Insect metamorphosis constitutes an evolutionarily successful ecological adaptation strategy that achieves temporal niche partitioning, mitigates predator pressure and seasonal constraints, and enables developmental plasticity in response to environmental variables (e.g., photoperiod, temperature, and food availability), thereby enhancing organismal fitness across heterogeneous habitats. As the most vulnerable phase in an insect’s life cycle, metamorphosis is regulated by a complex interplay of genetic, hormonal, and environmental factors. The precise hormonal regulation of metamorphosis, particularly through the juvenile hormone (JH)-ecdysone signaling cascade, presents a potential target for developing next-generation, eco-conscious insect control strategies.

The silkworm (*B. mori*) serves as both a holometabolous lepidopteran model organism and an economically significant species. During metamorphosis, larval tissue degradation occurs primarily through programmed cell death (PCD) mechanisms, particularly apoptosis and autophagy, which mediate the elimination of senescent tissues [1,2,3]. This developmental process is coordinately regulated by 20E through its induction of a downstream transcriptional cascade [4,5,6,7]. The ecdysone signaling pathway primarily upregulates autophagy-related genes, including the Atg family [8], *E74*, and *Met*, etc., while suppressing mTOR activity to induce autophagy. Simultaneously, 20E upregulates pro-apoptotic genes (including *Apaf-1*, *Nedd2 like1*, *ICE1*, *Arp*, and *IAP*), thereby triggering PCD in the fat body during larval–pupal metamorphosis [9]. These lines of evidences demonstrate that 20E-mediated apoptosis and autophagy constitute essential mechanisms for larval tissue degradation during metamorphosis. However, the insufficient elucidation of 20E’s molecular mechanisms remains a major barrier to developing targeted, 20E-based insect control strategies. Research on epigenetic-based gene regulation has revealed diverse post-translational modifications (PTMs), including methylation, acetylation, phosphorylation, and ubiquitination, that critically modulate gene expression. These regulatory mechanisms are essential for proper animal development and tissue homeostasis [10,11], with demonstrated roles in 20E-mediated gene expression during insect metamorphosis. Notably, the acetylation/deacetylation of Atg proteins regulate autophagy in silkworm cells [3], while ubiquitination and SUMOylation coordinate 20E-dependent developmental transitions during larval metamorphosis [10]. While the role of ubiquitin modification in apoptosis regulation is well established [12], its specific function in 20E-mediated apoptotic pathways within metabolic tissues remains poorly characterized.

The *p53* gene represents a crucial tumor suppressor conserved across metazoans [13,14]. Structural and functional analyses reveal that invertebrate *p53* homologs, including insect variants, maintain significant similarity with their vertebrate counterparts [15], with conserved mechanistic pathways [16,17]. In *B. mori*, *Bmp53* has been identified as a vertebrate p53 ortholog and characterized as a putative apoptosis regulator [18]. Our previous research has demonstrated that *Bmp53* can induce apoptosis in silkworm cells and promote the metamorphosis of insect pupae through this effect. Furthermore, we analyzed and predicted the apoptotic regulatory mode of Bmp53, which includes the ubiquitinating structural protein Mdm2-like [19]. In vertebrates, Mdm2 (mouse double minute 2) serves as the primary negative regulator of p53, mediating ubiquitination and degradation to maintain protein stability [20,21]. While most invertebrates, including *Drosophila*, lack an *Mdm2* ortholog [22], the *Drosophila* p53 stabilizer, Corp (a companion of reaper), shows partial sequence homology with MDM2 [23,24]. However, Corp lacks E3 ubiquitin ligase activity, while our integrated yeast two-hybrid and high-throughput sequencing platform identified MDM2-like as a functional ubiquitin ligase regulator of Bmp53 [19]. The structural analysis revealed a striking conservation of human MDM2 [25,26], suggesting that the ubiquitination regulation of Bmp53 may share greater mechanistic similarity with vertebrate systems than with other insect models like *Drosophila*.

Our study shows that Mdm2-like protein mediates Bmp53 ubiquitination, significantly attenuating 20E-dependent apoptotic signaling compared to Bmp53 overexpression alone. These findings establish Bmp53 ubiquitination as a regulatory component of 20E-mediated apoptosis. In other words, we identify the Mdm2-like/Bmp53 ubiquitination pathway as a novel apoptotic regulation mechanism in *B. mori*, with potential conservation across *Lepidoptera*, where 20E serves as a critical regulatory factor. These results reveal the role of a novel post-translational modification pathway in the 20E-mediated apoptosis signaling pathway of silkworms, providing a theoretical reference for the development of hormone-based eco-friendly pest control strategies.

## 2. Materials and Methods

### 2.1. Reagents and Antibodies

The reagents used in this study were obtained from the following suppliers: 4% Paraformaldehyde Fix Solution (Sangon, Shanghai, China; E672002); BSA (Beyotime, Shanghai, China; ST023); Triton X-100 (Sangon, Shanghai, China; A417820); DAPI (Solarbio, Beijing, China; C0065); One Step Western Kit HRP (Mouse) (CWBio, Taizhou, China; CW2030M); One Step Western Kit HRP (Rabbit) (CWBio, Taizhou, China; CW2029M); Nuclear and Cytoplasmic Extraction Kit (CWBio, Taizhou, China; CW0199S); Protease Inhibitor Cocktail (CWBio, Taizhou, China; CW2200S); DAB Kit (CWBio, Taizhou, China; CW0125M); Protein A/G Resin (Transgen, Beijing, China; DP501); HiFiScript gDNA Removal RT MasterMix (CWBio, Taizhou, China; CW2020M); ChamQ SYBR qPCR Mater Mix (Vazyme, Nanjing, China; Q311); Annexin V-EGFP/PI Cell Apoptosis Detection Kit (Transgen, Beijing, China; FA111); TUNEL BrightRed Apoptosis Detection Kit (Vazyme, Nanjing, China; A113); MG132 (Med Chem Express, New Jersey, USA; HY-13259); 20E was obtained from Zhenjiang Zhongnong Biotechnology Co., Ltd. (Zhenjiang, China). All experiments were performed according to the manufacturers’ protocols. The antibodies used in this study for co-immunoprecipitation and Western blot are as follows: rabbit anti-V5 (Huabio, Hangzhou, China; ET1605-41); rabbit anti-Flag (Sigma-Aldrich, Darmstadt, Germany; F7425-2MG); rabbit anti-ubiquitin (Boster, Wuhan, China; PB9122); iFluorTM 488 goat anti-rabbit IgG H&L (Huabio, Hangzhou, China; HA1121).

### 2.2. Plasmid Construction

pIZ-V5-His, pIZ-EGFP-V5-His, pIZ-EGFP-Bmp53-V5-His [19], and pIZ-mCherry-V5-His were preserved in our laboratory. The 3xFlag tag of pIZ-3xFlag was synthesized by Sangon Biotech (Shanghai, China) and constructed by inserting 3xFlag sequences into *Sac*II and *Age*I cleavage sites on the basis of pIZ-V5-His. The pIZ-Bmp53-3xFlag fusion plasmid was constructed by inserting cloned *Bmp53* into the *Bam*HI and *Xho*I sites of the pIZ-3xFlag vector; the amplified Bmp53 ORF was similarly cloned using Bmp53-F and Bmp53-R(CC) primers [19]. pIZ-Mdm2-like-V5-His fusion plasmid was constructed by inserting cloned *Mdm2-like* into the *Eco*RI and *Xho*I sites of pIZ-V5-His vector, and the cloning primers were Mdm2-CF: CCGGAATTCATGAACACAACATTTTG and Mdm2-CR: ATACTCGAGTAGCACGACGGCGCGC. The pIZ-mCherry-Mdm2-like-V5-His plasmid was constructed by cloning Mdm2-like into the EcoRI and XhoI sites of the pIZ-mCherry-V5-His vector, using the primers Mdm2-MF (CCGGAATTCaATGAACACAACATTTTGT) and Mdm2-MR (ATActcgagAATAGCACGACGGCGCGC).

### 2.3. Cell Culture and Treatment

The *B. mori* ovary epithelial cell line (BmN) was preserved in our laboratory and cultured in TC-100 medium, supplemented with 10% fatal bovine serum (FBS) and incubated at 28 °C.

Cells were transfected with the above plasmids using Entranster-H4000 transfection reagent (Engreen, Beijing, China), according to the manufacturer’s instructions. For drug treatment, MG132 (5 µM) or 20-Hydroxyecdysone (20E) (50 ng/µL) were added, whilst the negative control group received an equal volume of DMSO or 20% ethyl alcohol.

### 2.4. Ubiquitination Detection by Fluorescence Mocroscopy and Western Blotting

To demonstrate the E3 ubiquitin ligase activity of Mdm2-like, confluent BmN cells were subcultured at a 1:2.5 ratio. After 24 h, cells in six-well plates were transfected with 2 μg of either pIZ-Mdm2-like-V5-His or pIZ-V5-His plasmid (empty vector control) using Entranster™-H4000 transfection reagent. Untransfected BmN cells served as mock controls. Cells were harvested 48 h post-transfection for subsequent analysis. To assess ubiquitination levels mediated by Mdm2-like and Bmp53 interaction, BmN cells in six-well plates were co-transfected with 2 μg each of pIZ-Mdm2-like-V5-His and pIZ-Bmp53-3xFlag plasmids. Control transfections containing 2 μg each of empty vectors (pIZ-V5-His + pIZ-3xFlag) were performed in parallel. All transfections used the same subculture method and Entranster™-H4000 reagent as previously described. Cells were harvested 48 h post-transfection for ubiquitination assays. Then, cells were fixed with 4% paraformaldehyde solution and permeabilized with 0.25% TritonX-100 in PBS for 10 min, and then blocked with 5% BSA for 15 min at room temperature. Cells were probed with the rabbit anti-ubiquitin antibody at 1/200 dilution for overnight at 4 °C. iFluorTM 488 goat anti-rabbit IgG H&L was used as the secondary antibody at 1/500 dilution. DAPI was used as nuclear counterstain. Finally, the fluorescence signal was detected by the fluorescent microscope (Olympus Corporation, Tokyo, Japan, IX83) and confocal microscope (Leica, Wetzlar, Germany, SP8). Fluorescent cell images were processed using Adobe Photoshop (v24.2) (Adobe, San Jose, CA, USA).

BmN cells in 10 cm plates were transfected with 5 μg pIZ-Mdm2-like-V5-His plasmid for 48 h. Nucleoproteins were extracted using a Nuclear and Cytoplasmic Extraction Kit (CWBio, CW0199S) and incubated with rabbit anti-V5 antibody (Huabio, ET1605-41, 1:200 dilution) for 2–4 h at 4 °C. Protein A/G agarose beads (Beyotime, P2055, China) were added and incubated overnight at 4 °C. Beads were pelleted (1000× *g*, 5 min), washed twice with PBS, and resuspended in 5× protein loading buffer (Beyotime, P0015L, China). For controls, anti-V5 was replaced with species-matched IgG. Samples were denatured (95 °C, 5 min), resolved by 10% SDS-PAGE, and transferred to PVDF membranes. Ubiquitinated proteins were detected using rabbit anti-ubiquitin antibody followed by HRP/Rabbit secondary antibodies.

### 2.5. Co-Immunoprecipitation (Co-IP)

Nuclear protein lysates were extracted from BmN cells using a nucleoprotein extraction kit containing protease inhibitors. The lysate was incubated overnight at 4 °C with 2 µL antibody (rabbit anti-V5 or rabbit anti-Flag). The protein–antibody complex was then mixed with protein A/G agarose beads and incubated for 2 h at 4 °C. After incubation, the beads were washed with PBS, resuspended, and boiled in protein loading buffer. The supernatant was collected by centrifugation for SDS-PAGE analysis. Following electrophoresis, proteins were transferred to a PVDF membrane and detected by immunoblotting.

### 2.6. Real-Time Quantitative Polymerase Chain Reaction (RT-qPCR)

Total RNA was extracted from cells TRIzol Reagent followed by DNase I treatment (RNase-free) to remove genomic DNA contamination. The first strand cDNA was synthesized from 1 μg total RNA using a HiFiScript gDNA Removal cDNA Synthesis Kit(CWBio, Taizhou, China; CW2020M) according to the manufacturer’s protocol. Quantitative PCR was performed with ChamQ SYBR qPCR Mater Mix using 100 ng cDNA template per reaction. RP49 served as the internal reference gene. The gene-specific primer sequences are provided in Table 1.

### 2.7. Flow Cytometry

Apoptosis was assessed using an Annexin V-EGFP/PI Cell Apoptosis Detection Kit (Transgen, Beijing, China; FA111) following the manufacturer’s protocol. Briefly, harvested cells were washed twice with cold PBS and resuspended in 1 × binding buffer. Cells were simultaneously stained with Annexin V-EGFP and propidium iodide (PI) (1:1 *v*/*v*) and incubated on ice for 15 min in the dark. Samples were analyzed immediately using a BD FACSVerse flow cytometer (BD Biosciences, New York, NY, USA) with fluorescence detected through FL1 (EGFP) and FL3 (PI) channels. Data were processed using FlowJo software (v10.8.1, BD Life Sciences, Ashland, Oregon, USA;).

### 2.8. TUNEL Assay

Following PBS washes, the cells were fixed with 4% paraformaldehyde for 30 min and permeabilized with 0.25% Triton X-100 for 30 min. After blocking with 5% BSA for 1 h, the cells were incubated with TUNEL solution for 1 h at 37 °C in a humidified chamber. Nuclei were counterstained with DAPI (1 μg/mL) for 5 min. Fluorescent images were captured using an inverted microscope (IX83, Olympus, Japan) equipped with appropriate filter sets for DAPI and TUNEL detection.

### 2.9. Statistics

All experiments were performed in triplicate with at least three independent biological replicates. Data are presented as mean ± standard deviation (SD). Statistical analyses were conducted using GraphPad Prism 8, with multiple group comparisons evaluated by one-way ANOVA followed by Tukey’s post hoc test, and pairwise comparisons analyzed by two-tailed Student’s *t*-test. A threshold of *p* < 0.05 was considered statistically significant.

## 3. Results

### 3.1. Mdm2-like Is a Ubiquitinating Enzyme

**Figure 1:**
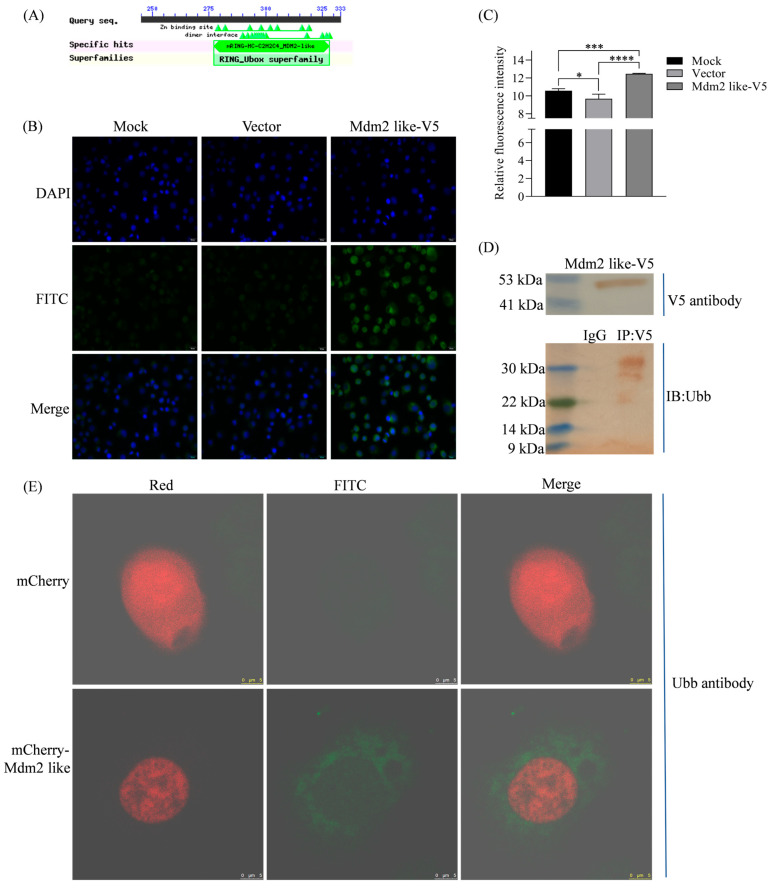
Structural prediction and functional analysis of Mdm2-like ubiquitin ligase activity. (**A**) Domain architecture prediction of Mdm2-like using NCBI’s Conserved Domain Database (https://www.ncbi.nlm.nih.gov/Structure/cdd/wrpsb.cgi (accessed on 11 March 2023)) revealed a RING finger/U-box domain. RING finger domains typically function as E3 ubiquitin ligases, facilitating ubiquitin transfer from E2 conjugating enzymes to substrate proteins. The predicted structure suggests that Mdm2-like may operate as an E3 ubiquitin-protein ligase. (**B**) Ubiquitination assay in Mdm2-like-overexpressing cells. Ubiquitin signals (green) were detected using Ubb antibody with iFluor™ 488-conjugated secondary antibody. Nuclei were counterstained with DAPI (blue). Scale bar: 20 μm (40× magnification). (**C**) Quantification of ubiquitin fluorescence intensity from panel B. Data represent mean ± SD from three independent experiments (* *p* < 0.05, *** *p* < 0.005 **** *p* < 0.0001 by Student’s *t*-test). (**D**) Western blot analysis of ubiquitinated proteins in BmN cells transfected with pIZ-Mdm2-like-V5-His. Immunoprecipitation (IP) was performed with rabbit anti-V5 antibody, followed by immunoblotting (IB) with rabbit anti-ubiquitin antibody. (**E**) Cells expressing the mCherry-Mdm2-like fusion protein exhibited significantly enhanced ubiquitination signals compared to controls. Ubiquitin signals (green) were detected using rabbit anti-ubiquitin antibody with iFluorTM 488 goat anti-rabbit IgG H&L antibody.

Mdm2-like is a *B. mori* homolog of Mdm2, with a predicted molecular weight of 36.7 kDa. Structural analysis revealed that Mdm2-like contains a RING finger/U-box domain (Figure 1A). RING finger/U-box proteins are a diverse family of proteins involved in critical cellular processes such as tumorigenesis, viral replication, cell cycle regulation, and apoptosis. Most proteins containing this domain function as ubiquitin ligases, suggesting that Mdm2-like participates in ubiquitination. Additionally, while there is no clearly defined p53 binding domain, the Mdm2-like region (residues 55–113) serves as a secondary binding site for p53 [25,26], suggesting potential physical interaction with Bmp53. Cellular analysis using Ubiquitin (Ubb) antibody and iFluor™ 488-conjugated secondary antibody (Huabio, Hangzhou, China; HA1121) revealed significantly enhanced fluorescence signals in Mdm2-like overexpressing BmN cells (Figure 1B,C,E), with corresponding ubiquitination bands detected by Western blot (Figure 1D). These results validate the structural prediction and demonstrate that Mdm2-like possesses ubiquitin ligase activity.

### 3.2. Protein Interaction Between Mdm2-like and Bmp53

**Figure 2:**
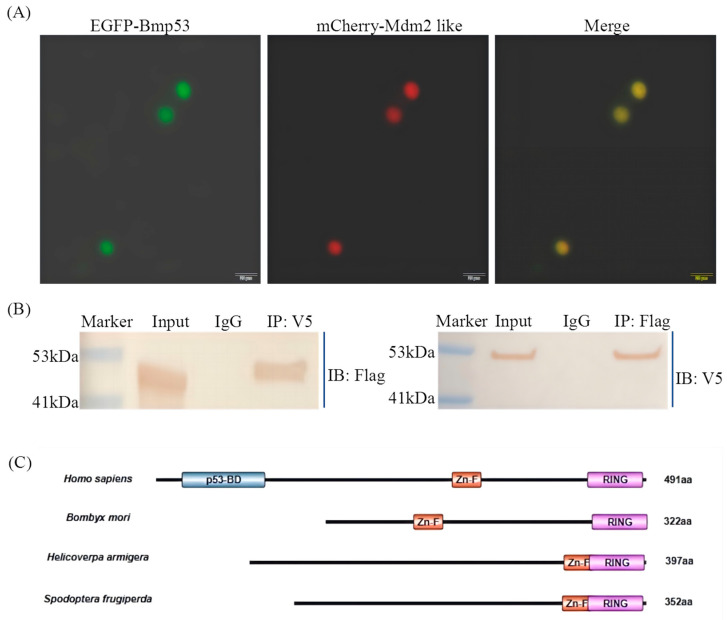
Interaction analysis between Mdm2-like and Bmp53. (**A**) Nuclear colocalization of EGFP-Bmp53 (green) and mCherry-Mdm2-like (red) in BmN cells. Scale bar: 20 μm. (**B**) Co-immunoprecipitation (Co-IP) assay validating the Mdm2-like–Bmp53 interaction. BmN cells were co-transfected with pIZ-Bmp53-3xFlag and pIZ-Mdm2-like-V5-His plasmids. For reciprocal co-immunoprecipitation, proteins were pulled down using either anti-V5 (**left**) or anti-Flag antibodies (**right**), followed by immunoblotting with anti-Flag or anti-V5 antibodies, respectively. Control lanes show IgG immunoprecipitation. IB, immunoblot; IP, immunoprecipitation. (**C**) Comparative analysis of *B. mori*, *Helicoverpa armigera*, and *Spodoptera frugiperda* reveals shared lepidopteran-specific features, including a characteristic ring region and conserved zinc finger motifs.

Our prior work predicted that *Bmp53* in *B. mori* may be regulated through novel ubiquitination-dependent apoptotic mechanisms that diverge from those observed in model insects such as *Drosophila melanogaster*. To test whether the ubiquitin ligase Mdm2-like participates in this regulation, we examined their physical interaction. The co-expression of EGFP-Bmp53 and mCherry-Mdm2-like fusion proteins revealed the nuclear colocalization of Bmp53 and Mdm2-like, suggesting a potential interaction in the nucleus (Figure 2A). We co-expressed Flag-tagged Bmp53 with V5/His-tagged Mdm2-like in BmN cells and performed co-immunoprecipitation (Co-IP) assays using tag-specific antibodies (Figure 2B). The successful pull-down of both proteins demonstrates a direct interaction, suggesting that Mdm2-like may regulate Bmp53 through ubiquitination in silkworms, and MDM2 proteins across *Lepidoptera* species exhibit conserved structural homology while lacking the canonical p53-binding domain found in vertebrates (Figure 2C), potentially representing a *Lepidoptera* species-specific apoptotic control mechanism.

### 3.3. Mdm2-like Inhibits Bmp53-Mediated Apoptosis Through Ubiquitination

**Figure 3:**
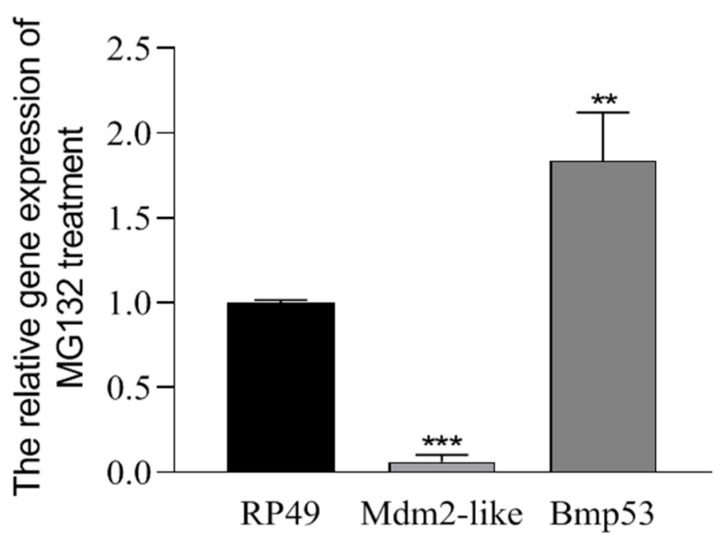
Effect of proteasome inhibition on *Mdm2-like* and *Bmp53* expression. The BmN cells were treated with either 5μM MG132 or DMSO (vechicle control) for 12 h. Relative mRNA expression levels of *Mdm2-like* and *Bmp53* were quantified by qRT-PCR using the 2 ^−ΔΔCT^ method. Data represent mean ± SD from three biological replicates (each with three technical replicates). Statistical significance was determined by Student’s *t*-test (** *p* < 0.01, *** *p* < 0.001).

**Figure 4:**
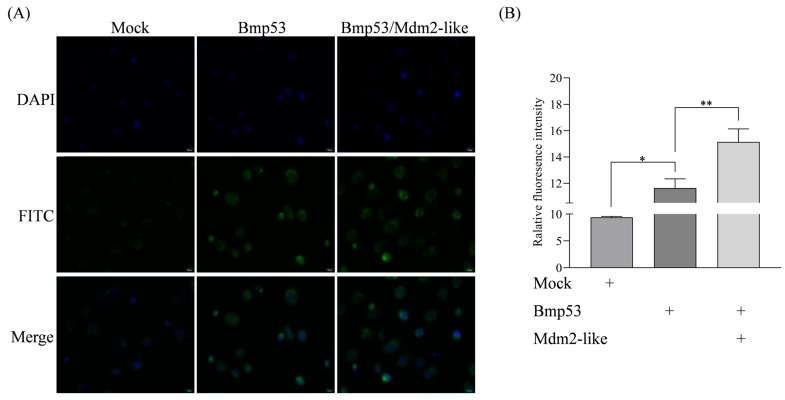
Enhanced ubiquitination in BmN cells co-expressing Bmp53 and Mdm2-like. (**A**) Detection of the ubiquitination level in BmN cells overexpressing either Mdm2-like alone or both Bmp53 and Mdm2-like. Ubiquitin signals (green) were detected using rabbit anti-ubiquitin antibody with iFluorTM 488 goat anti-rabbit IgG H&L. Nuclei were counterstained with DAPI (blue). Scale bar: 20 μm (40× magnification); (**B**) quantification of relative ubiquitin fluorescence intensity from panel (**A**). Data represent mean ± SD from three independent biological replicates (* *p* < 0.05, ** *p* < 0.01 by Student’s *t*-test).

**Figure 5:**
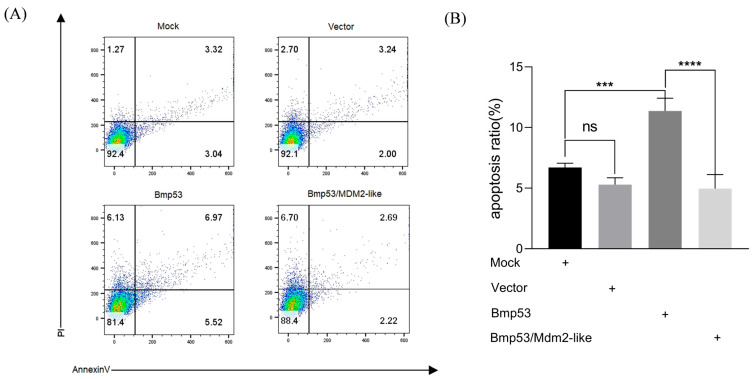
Mdm2-like suppresses Bmp53-induced apoptosis in BmN cells. (**A**) Representative flow cytometry plots of apoptotic BmN cells 48 h after transfection with the following: pIZ-Bmp53-3XFlag (Bmp53 overexpression), pIZ-Mdm2-like-V5-His (Mdm2-like overexpression), both plasmids (co-expression). Mock: untransfected control cells. (**B**) Quantitative analysis of apoptosis rates. Data represent mean ± SD from three independent experiments (*** *p* < 0.001, **** *p* < 0.0001 by Student’s *t*-test; ns, not significant).

While the interaction between Mdm2-like and Bmp53 has been established, the functional consequences of this interaction remain unclear. MG132 is a protease inhibitor that specifically targets the 26S proteasome in the ubiquitin–proteasome system (UPS). The UPS mediates the degradation of ubiquitinated proteins, thereby maintaining protein quality control and regulating cellular protein levels [27]. By inhibiting proteasomal activity, MG132 causes accumulation of ubiquitinated proteins within cells and induces apoptosis [28]. Our findings demonstrate that MG132, a ubiquitination inhibitor, significantly suppresses Mdm2-like while upregulating Bmp53 expression (Figure 3), suggesting reciprocal regulation through the ubiquitin–proteasome pathway. To further characterize this relationship, we quantified ubiquitination levels in transfected cells using immunofluorescence with anti-ubiquitin (Ubb) antibody and an iFluor™ 488 green fluorescent secondary antibody. Our data demonstrate a functional interplay where Mdm2-like counteracts Bmp53’s pro-apoptotic activity via ubiquitination. This regulatory mechanism is evidenced by the following: (1) enhanced ubiquitin signals in co-expressing cells (Figure 4) and (2) a significant reduction in apoptosis when Mdm2-like is present (Figure 5). The results indicate that Mdm2-like functions as a crucial negative regulator of Bmp53-induced apoptosis in silkworm cells via its ubiquitin ligase activity.

### 3.4. Mdm2-like Is Involved in 20E-Mediated Apoptosis Signaling Pathway Regulation via Ubiquitinating Bmp53

**Figure 6:**
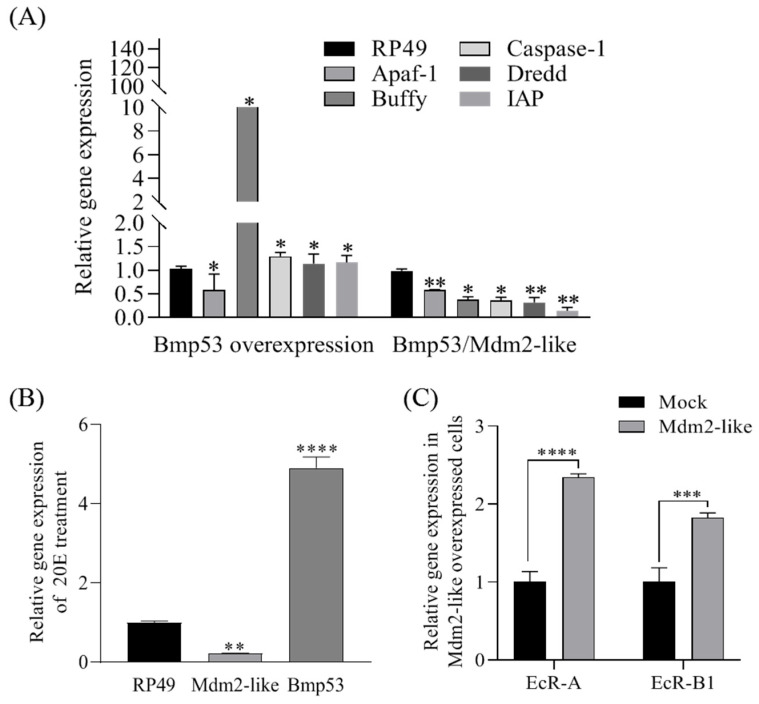
Role of the Mdm2-like–Bmp53 axis in 20E-regulated apoptosis signaling pathway. (**A**) Apotosis-related gene expression profiles folloing Bmp53 or Bmp53/Mdm2-like overexpression in BmN cells; (**B**) transcript levels of *Bmp53* and *Mdm2-like* in response to 10μM 20E treatment for 12 h; (**C**) effect of Mdm2-like overexpression on ecdysone receptor genes (*EcR-A* and *EcR-B1*). Data represent mean ± SD from three biological replicates (each with three technical replicates). Statistical significance was determined by Student’s *t*-test (* *p* < 0.05, ** *p* < 0.01, *** *p* < 0.001, **** *p* < 0.0001).

The overexpression of *Bmp53* in BmN cells significantly altered the expression of key apoptosis-related genes in the 20E signaling pathway, upregulating *Caspase1*, *IAP*, *Dredd*, and *Buffy*, while markedly downregulating *Apaf-1 (*Figure 6A). Strikingly, the co-expression of the ubiquitin ligase Mdm2-like with Bmp53 reversed these expression changes (except for *Apaf-1*), demonstrating Mdm2-like’s antagonistic role in Bmp53-mediated apoptosis regulation. We further observed that 20E treatment (10 μM, 12 h) upregulated *Bmp53* while downregulating *Mdm2-like* expression (Figure 6B). Intriguingly, Mdm2-like overexpression increased the transcript levels of ecdysone receptor genes *EcR-A* and *EcR-B1* (Figure 6C), suggesting a potential negative feedback loop between 20E signaling and Mdm2-like activity.

**Figure 7:**
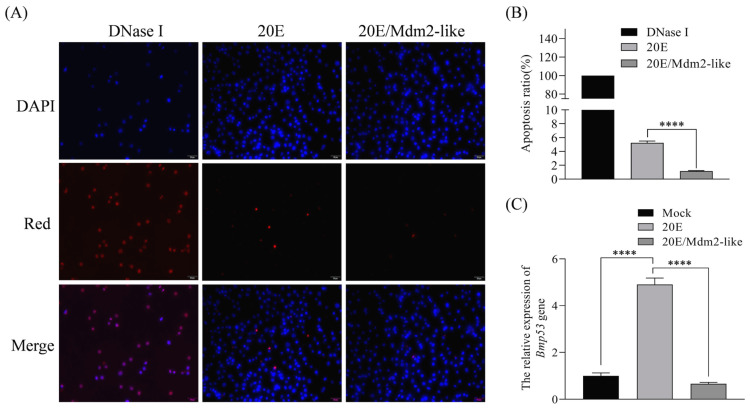
Mdm2-like modulates 20E-induced apoptosis in BmN cells. (**A**) TUNEL assay detecting apoptosis and DNA damage in the following: positive control cells (Dnase I treatment,), cells treated with 50 μM 20E, cells overexpressing Mdm2-like + 50 μM 20E treatment (scale bar: 50 μm); **(B)** quantification of TUNEL-positive cells from panel A. Data normalized to positive controls; **(C)** relative *Bmp53* expression levels under different treatment conditions. All data represent mean ± SD from three independent experiments (**** *p* < 0.0001).

The 20E plays well-characterized roles in coordinating apoptosis and autophagy during insect metamorphosis. Our study reveals that Mdm2-like protein antagonizes 20E-induced apoptosis and significantly downregulates *Bmp53* expression (Figure 7C). 20E reciprocally suppresses Mdm2-like transcription (Figure 6B) and Mdm2-like overexpression mitigates 20E-mediated apoptotic effects (Figure 7A,B). This regulatory network parallels the evolutionarily conserved Mdm2–p53 axis in vertebrates, where Mdm2 maintains p53 homeostasis through ubiquitin-mediated degradation. Our findings establish that the 20E–Mdm2-like–Bmp53 axis forms a dynamic equilibrium that may be able to precisely orchestrate tissue remodeling during silkworm metamorphosis.

## 4. Discussion

Ubiquitination is a key post-translational modification that modulates the stability and function of proteins critical for cell growth, proliferation, and survival [29]. Notably, ubiquitination plays a pivotal role in the regulation of cell death, which is essential for animal development as well as multi-tissue physiology and pathology [10,12]. Our prior work revealed that the *Bmp53* gene, which is a key regulator of silkworm cell apoptosis and larval–pupal metamorphosis [19], is likely under the direct control of ubiquitination enzymes.

*Bmp53* is a homolog of vertebrate p53 [18]. The p53 protein plays a crucial role in the apoptotic signaling pathway induced by DNA damage, and its levels are meticulously regulated within the cell [30,31], though its regulatory mechanisms in invertebrates remain less defined. In vertebrates, p53 stability is tightly governed by ubiquitination, primarily mediated by the E3 ligase Mdm2, which targets p53 for proteasomal degradation to fine-tune apoptotic responses [20,23]. Given this conserved role of ubiquitination in p53 regulation, we hypothesize that Bmp53 may similarly be modulated by ubiquitin-dependent pathways during silkworm development. Notably, some invertebrates, such as *Drosophila*, lack a homolog of Mdm2 [22]. Instead, the *Drosophila* protein Corp shares sequence homology with Mdm2 and stabilizes Dp53 [23,24]. However, Corp lacks E3 ubiquitin ligase activity, implying divergent regulatory mechanisms between insects and vertebrates. This study demonstrates that an Mdm2-like protein in silkworms possesses intrinsic ubiquitination activity (Figure 1) and directly interacts with Bmp53 to suppress its pro-apoptotic function (Figure 2, Figure 3, Figure 4 and Figure 5). Notably, Mdm2-like lacks the p53-binding domain found in its mammalian homolog, which aligns with previous findings [25,26]. Although we could not detect its physical interaction with Bmp53 in whole-cell lysates, subsequent nucleoprotein extraction and testing confirmed their binding. We speculate that this interaction is tightly regulated in a spatiotemporal manner, though the underlying mechanism warrants further investigation. Regardless, these results establish the Mdm2-like–Bmp53 axis as a critical ubiquitination-mediated regulatory mechanism controlling apoptosis in silkworm cells, revealing functional conservation with vertebrate p53 regulation while highlighting insect-specific adaptations. Furthermore, the observed ubiquitination levels upon Mdm2 overexpression, combined with Bmp53’s predicted apoptotic regulatory role, suggest that Mdm2-like proteins likely target additional factors beyond Bmp53 through ubiquitination. In future studies, we will identify and characterize other Mdm2-like regulatory proteins to comprehensively elucidate the ubiquitination-mediated control of apoptosis in silkworms.

During insect metamorphosis, senescent larval tissues are eliminated through programmed cell death (PCD), primarily via apoptosis and autophagy, a process mediated by ecdysone (20-hydroxyecdysone, 20E) and its triggered transcriptional cascades [6,9]. 20E exerts its biological function by targeting specific nuclear receptors, primarily the ecdysone receptor (EcR). Upon forming a complex with its heterodimeric partner EcR-USP, 20E triggers the rapid and robust induction of key response genes [32]. During the larval–pupal transition in *Bombyx mori*, 20E upregulates multiple apoptosis-related genes, including *Apaf-1*, *Nedd2-like1*, *ICE1*, *Arp*, and *IAP*, thereby promoting fat body apoptosis [9]. Our study reveals that Mdm2-like proteins regulate 20E-mediated apoptosis signaling pathway through Bmp53 ubiquitination. As 20E is a critical hormone governing insect metamorphosis and development, these findings establish a novel link between ubiquitination-dependent protein control and the hormonal regulation of programmed cell death in silkworms. Our current understanding of the ubiquitination axis in metagenesis and 20E signaling is largely based on prior findings [19]. To further validate this, we performed genetic knockout experiments targeting MDM2-like and Bmp53. However, the high lethality of homozygous mutants has so far prevented the establishment of stable lines. We acknowledge two major challenges: (1) the inherent complexity of 20E pathway regulation [6] and (2) the difficulty in isolating direct phenotypic effects attributable to the MDM2-like–Bmp53 axis. While we regret the inability to provide organism-level evidence for this mechanism at present, it remains a key focus of our ongoing experimental optimization efforts.

Studies on epigenetic-based gene regulation have unveiled various post-translational modification mechanisms (PTMs), including methylation, acetylation, phosphorylation, and ubiquitination, as well as 20-hydroxyecdysone (20E)-induced gene expression regulation. For example, the acetylation and deacetylation of Atg proteins critically regulate autophagy in silkworm cells [3], whereas ubiquitination and SUMOylation modulate 20E-dependent larval development [10]. While the involvement of ubiquitination in apoptosis regulation is well-established, its role in modulating 20E-dependent apoptosis during metamorphic tissue remodeling remains poorly characterized. In conclusion, our findings provide foundational insights into the regulation of silkworm metamorphosis. We demonstrate that Bmp53 promotes developmental progression in *B*. *mori*, while Mdm2-mediated ubiquitination suppresses *Bmp53* expression and its pro-apoptotic activity under 20E regulation. Importantly, we identify a putative 20E–Mdm2-like–Bmp53 regulatory axis that maintains a dynamic balance to regulate cell apoptosis. This newly discovered mechanism represents a crucial area for future investigation in insect developmental biology.

## 5. Conclusions

**Figure 8:**
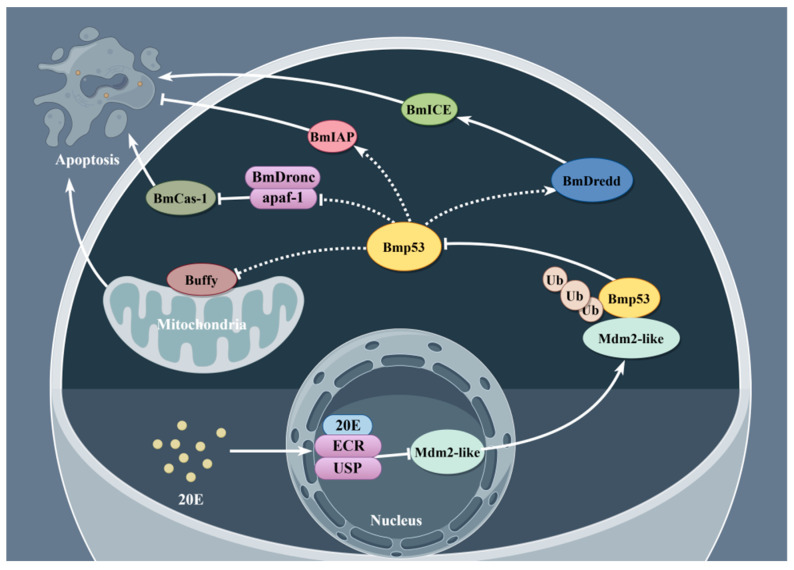
Schematic representation of the 20E–Mdm2-like–Bmp53 ubiquitination regulatory axis (created with Figdraw: https://www.figdraw.com (accessed on 18 July 2024)).

In summary (Figure 8), our findings demonstrate that Mdm2-like directly participates in silkworm cells apoptosis through the ubiquitination-mediated regulation of Bmp53. This study reveals, for the first time, the Mdm2-like/Bmp53 ubiquitination pathway as a novel apoptotic regulatory mechanism in *B*. *mori*, with potential implications for other lepidopteran species. Furthermore, this study underscores the critical involvement of the ecdysone signaling molecule 20E in this regulatory pathway. These findings establish a foundation for future research on ubiquitination-mediated control of 20E-dependent metamorphosis and developmental processes. Previous studies have established that the *Bmp53* gene plays a pivotal role in larval–pupal metamorphosis in *B*. *mori*. Our findings significantly advance the understanding of the genetic regulatory mechanisms governing tissue remodeling during lepidopteran metamorphosis. Moreover, these insights establish a theoretical reference for developing eco-friendly pest control strategies through the targeted disruption of protein modification pathways, thereby contributing to sustainable lepidopteran pest management.

This study provides compelling evidence for the following: Mdm2-like exhibits intrinsic ubiquitin ligase activity and physically interacts with Bmp53 to suppress its pro-apoptotic function; 20E serves as a critical regulator of the Mdm2-like/Bmp53 ubiquitination pathway.

## Figures and Tables

**Table 1:** Primers used for RT-qPCR.

Name	Sequence
RP49-F	AGGCATCAATCGCATCGCTATG
RP49-R	TTGTGAACTAGGACCTTACGGAATC
Bmp53-F	CATCTTCACCCTGGAGAGCG
Bmp53-R	GCTCCGATAATTTCCAGCGG
Mdm2-F	GGCCGAAACGTAAACGCAAA
Mdm2-like-R	AGCGCTACTGTCAACGTCTC
Apaf1-F	TGGTCACGATGGCTTCAGAG
Apaf1-R	AGCCGCTTAAAAACAAGGTAGA
Buffy-F	TCAGCTATGCTACGCTCAGACA
Buffy-R	ATCCATGATCCAGGCTCCTC
Caspase1-F	AAACGGCAACGAAGACGAAG
Caspase1-F	TGCCCGTGCGAGATTTTAGAT
Dredd-F	TACTGGGCAACAGCACCT
Dredd-R	ATGGGAACCTGAGGATGA
IAP-F	TGACGAAAGTTGCTAAAAATGGA
IAP-R	GGTGACGGGAGTGTGGATGT
BmEcR-AF	GCTTCAAGCATAAATGGC
BmEcR-AR	TCAGTGCGTTGTAGTGGTAT
BmEcR-B1F	AGCAGCCGTCGCTGGTAGAA
BmEcR-B1R	TCGGTCAGCACGCTCAGGAT

## Data Availability

The original contributions presented in this study are included in the article. Further inquiries can be directed to the corresponding author.
